# Laboratory Diagnostics of Aspergillosis: Present State and Future Directions

**DOI:** 10.3390/jof12050379

**Published:** 2026-05-21

**Authors:** Rok Tomazin, Tadeja Matos

**Affiliations:** Institute of Microbiology and Immunology, Faculty of Medicine, University of Ljubljana, SI-1000 Ljubljana, Slovenia; rok.tomazin@mf.uni-lj.si

**Keywords:** aspergillosis, culture-based methods, galactomannan, PCR, laboratory diagnostics

## Abstract

Aspergillosis encompasses a heterogeneous spectrum of diseases caused by filamentous fungi of the genus Aspergillus, ranging from allergic airway disorders and chronic pulmonary infection to rapidly progressive invasive disease. *Aspergillus fumigatus* is the predominant pathogen worldwide, although other species, including *Aspergillus flavus*, *Aspergillus terreus* and cryptic species, contribute to morbidity and may exhibit intrinsic or acquired antifungal resistance. Early and accurate laboratory diagnosis is essential for timely treatment, appropriate antifungal selection, and stewardship. Traditional culture remains foundational, enabling confirmation of viable organisms, species-level identification, and antifungal susceptibility testing, but sensitivity is limited and turnaround times are prolonged. Non-culture approaches—including galactomannan, β-D-glucan, lateral flow assays, PCR, and next-generation sequencing—enhance diagnostic sensitivity, facilitate early detection, and allow identification of resistance-associated mutations. Optimal diagnostic performance is achieved through integrated, multimodal strategies combining laboratory tests with clinical and radiological findings. In invasive disease, concurrent use of biomarkers and molecular assays improves specificity and positive predictive value, while in allergic bronchopulmonary aspergillosis, immunological markers remain central. Future directions include standardised molecular protocols, novel antigenic and host-based biomarkers, and cost-effective, risk-adapted diagnostic algorithms to refine detection, guide therapy, and improve patient outcomes.

## 1. Introduction

Aspergillosis encompasses a heterogeneous spectrum of diseases caused by filamentous fungi of the genus Aspergillus, ranging from allergic airway disorders and chronic pulmonary infection to rapidly progressive, life-threatening invasive disease [1,2,3,4]. *Aspergillus fumigatus* is the predominant aetiological agent worldwide; however, other species, including *Aspergillus flavus*, *Aspergillus terreus*, and cryptic species within the *A. fumigatus* complex, also contribute substantially to morbidity and mortality and may exhibit intrinsic or acquired antifungal resistance [5,6]. Accurate laboratory identification is therefore essential not only for early diagnosis but also for appropriate antifungal selection and stewardship [7].

Globally, aspergillosis represents a substantial and often under-recognised public health burden. According to Denning et al. (2024) [8], invasive aspergillosis (IA) affects over 2.1 million individuals annually, resulting in approximately 1.8 million deaths. In addition, chronic pulmonary aspergillosis is estimated to affect more than 1.8 million people, with an annual mortality of 340,000 (18.5%), while allergic manifestations, including fungal asthma, impact around 11.5 million individuals worldwide and may contribute to approximately 46,000 asthma-related deaths each year. Despite advances in management, outcomes remain poor. Six-week mortality in IA ranges from 31% to 36%, particularly among patients with haematological malignancies or infections caused by azole-resistant strains [9]. The emergence and global spread of triazole resistance, especially in low- and middle-income countries, further complicates treatment and represents an increasing clinical challenge [10].

IA, particularly invasive pulmonary aspergillosis (IPA), predominantly affects patients with prolonged neutropenia, haematological malignancies, stem cell or solid organ transplantation, advanced chronic lung disease, or critical illness [1,11,12]. Mortality remains high, frequently exceeding 30–50% in high-risk populations [2,13,14]. Histopathological confirmation of tissue invasion and positive culture from sterile sites represent diagnostic gold standards, although both are limited by procedural invasiveness and suboptimal sensitivity [3,15,16].

Allergic bronchopulmonary aspergillosis (ABPA) is an immunologically mediated hypersensitivity disorder occurring mainly in patients with asthma or cystic fibrosis [2,5]. Diagnosis relies on a combination of clinical, radiological, and immunological criteria, including elevated total IgE and *Aspergillus*-specific IgE and IgG antibodies [2,17,18].

In recent decades, laboratory diagnostics have expanded beyond conventional microscopy and culture to include antigen detection, molecular assays, and next-generation sequencing (NGS) [16,19,20,21]. Each modality offers distinct advantages and limitations regarding sensitivity, specificity, turnaround time, and applicability across clinical contexts. Increasingly, integrated multimodal diagnostic strategies are recommended to enhance diagnostic accuracy and optimise antifungal therapy [2,7,13,22,23].

## 2. Culture-Based Methods and Antifungal Susceptibility Testing

### 2.1. Culture-Based Methods

Conventional fungal culture remains a fundamental component of aspergillosis diagnostics and continues to be included in the consensus definitions of the European Organisation for Research and Treatment of Cancer/Mycoses Study Group Education and Research Consortium (EORTC/MSGERC). Although non-culture methods have markedly improved early detection, culture retains several unique advantages: confirmation of viable organisms, species-level identification, and the possibility of performing antifungal susceptibility testing (AFST) [15,16,24,25].

#### 2.1.1. Diagnostic Yield and Interpretation

The sensitivity of culture in IPA is limited, often below 50%, especially in patients receiving mould-active prophylaxis or treatment. Culture results may take several days, and in some cases up to two weeks, depending on fungal growth kinetics. However, culture positivity from sterile tissue specimens remains highly specific and constitutes proof of invasive disease under current diagnostic definitions [11,16,26,27].

Interpretation of positive respiratory cultures is challenging. Isolation of *Aspergillus* spp. from respiratory samples—including sputum and tracheal aspirates—may indicate airway colonisation rather than invasive disease. This applies even to specimens from the lower respiratory tract, such as bronchoalveolar lavage fluid (BALF), as airway colonisation is common, particularly in patients with chronic obstructive pulmonary disease, bronchiectasis, or cystic fibrosis. Culture findings must therefore be interpreted in the context of host risk factors, radiological abnormalities, and adjunctive laboratory markers [4,12,13,28].

#### 2.1.2. Importance of Species-Level Identification

Species identification is clinically relevant for several reasons. Antifungal susceptibility varies between species; for example, *A. terreus* has intrinsic resistance to amphotericin B, making species recognition essential for appropriate therapeutic selection [24,29,30]. Additionally, azole resistance in *A. fumigatus*, often linked to environmental *cyp51A* mutations, has become a significant global clinical problem. Early detection of resistant strains allows timely modification of therapy and informs local antifungal stewardship strategies [7,31,32].

Cryptic species within the *A. fumigatus* complex (e.g., *Aspergillus lentulus*) may show reduced susceptibility to multiple antifungal classes and are often misidentified by conventional methods [24,29,30]. Accurate species delineation is therefore crucial for patient management, epidemiological surveillance, and infection control [2,7,30].

##### Role of Classical Morphological Identification

Despite the availability of molecular and proteomic methods, classical morphological identification remains the standard initial approach in many routine mycology laboratories [15,33,34,35,36]. Macroscopic colony characteristics (colour, texture, growth rate), combined with microscopic morphology (conidial heads, vesicle shape, phialide arrangement), allow identification to genus and often species level by experienced mycologists. Morphology-based methods are inexpensive, widely accessible, and require no specialised instrumentation, making them particularly valuable in resource-limited settings [13,15,35].

However, morphological identification has inherent limitations, as it requires experienced personnel with advanced mycological expertise; moreover, closely related or cryptic species may be phenotypically indistinguishable, and interobserver variability may occur [15,33,36,37]. Consequently, morphology is increasingly complemented by matrix-assisted laser desorption/ionisation time-of-flight mass spectrometry (MALDI-TOF MS), which provides rapid and accurate species-level identification when supported by comprehensive reference databases [33,38,39]. Molecular sequencing of ribosomal DNA or β-tubulin genes remains the reference method for definitive species identification in specialised laboratories [13,20,25,40,41].

In routine clinical practice, culture with morphological identification remains standard, supplemented by MALDI-TOF MS where available [13,15,25,39]. Advanced molecular identification and direct resistance detection from primary specimens currently remain largely confined to reference or research settings [7,20,42].

Culture-based diagnostics therefore remain indispensable despite their limited sensitivity and slower turnaround times [13,15,25]. They provide confirmation of viable infection, enable species-level identification essential for therapeutic decision-making, and allow antifungal susceptibility testing [24,29,30]. For these reasons, culture continues to represent a foundational element of integrated diagnostic strategies for aspergillosis.

## 3. Antifungal Susceptibility Testing

AFST is an essential component of aspergillosis diagnostics, especially in regions with emerging azole resistance or in cases of treatment failure or relapse, and is performed selectively based on clinical context and epidemiological considerations [24,29,30]. Amphotericin B, the triazoles (itraconazole, voriconazole, posaconazole, isavuconazole) and echinocandins (caspofungin, anidulafungin, micafungin) should be prioritised for AFST of clinically relevant *Aspergillus* spp., as these agents correspond directly to those used in routine clinical management. Although this selection reflects current evidence and treatment practice, therapeutic decisions must also consider PK/PD factors and clinical judgement beyond in vitro results [7,43,44].

Reference AFST is performed using standardised broth microdilution techniques as defined by the Clinical and Laboratory Standards Institute (CLSI) and the European Committee on Antimicrobial Susceptibility Testing (EUCAST). These methods determine minimum inhibitory concentration (MIC) values by exposing isolates to defined concentrations of antifungal agents under controlled laboratory conditions [24,45,46]. Interpretation relies on established clinical breakpoints and epidemiological cut-off values (ECOFFs), enabling distinction between wild-type and resistance-associated phenotypes [47,48].

Reference methodologies, although accurate, are technically demanding, time-consuming, and require specialised laboratory infrastructure and expertise [24,29,45,49]. Turnaround times may range from several days to over a week after culture recovery. Interpretation of MIC values may also differ between CLSI and EUCAST due to methodological differences, such as incubation conditions and growth endpoints. For example, CLSI uses a visual endpoint at 100% inhibition, whereas EUCAST employs a 50% growth reduction endpoint in many cases, which can result in discordant MIC results for certain antifungal–organism combinations [24,50,51].

Commercial gradient diffusion methods such as the Etest (bioMérieux) are often used in routine laboratories as practical alternatives [24,45,49]. Etest strips contain a predefined antifungal gradient on a plastic strip placed onto an inoculated agar plate; the resulting elliptical zone of inhibition intersects the strip at the MIC value after incubation. Etest offers ease of use and shorter turnaround times, but numerous studies have shown that MIC values for moulds may differ from reference broth microdilution results, with variable agreement across antifungal classes [46,49].

Commercial automated systems (e.g., Sensititre YeastOne, Micronaut-AM) provide ready-to-use microdilution panels covering multiple antifungal agents and have been validated against reference methods. These platforms reduce hands-on time and improve standardisation but are associated with higher costs and require incubation periods similar to reference techniques [45,49]. AFST is increasingly shifting from an optional test to a central component of personalised aspergillosis management. In clinical practice, commercial, standardised methods informed by CLSI or EUCAST guidelines are generally preferred, whereas the reference CLSI and EUCAST broth microdilution procedures are mainly applied in research, surveillance, and antifungal development [52,53].

Rapid phenotypic and molecular approaches, including direct detection of *cyp51A* mutations from clinical samples, enable timely, resistance-informed therapy. Emerging rapid colourimetric assays using metabolic indicators have potential to reduce turnaround times, though data for filamentous fungi, including *Aspergillus* spp., remain limited [24,45]. Regardless of method, AFST results must be interpreted in the context of clinical presentation, pharmacokinetics, and pharmacodynamics, with genotypic assays providing early indication of azole resistance to guide empirical therapy [24,29,49]. Integrating AFST with patient risk factors, therapeutic drug monitoring, and local epidemiology supports tailored antifungal selection and dosing, establishing AFST as a key tool in stewardship and surveillance strategies, particularly for invasive and chronic disease where resistance may emerge [31,32,42].

## 4. Antigen Testing: Galactomannan and Novel Biomarkers

Antigen detection is a central component of non-culture diagnostics for aspergillosis. These assays identify fungal cell wall components released during growth, providing evidence of active infection [13,19,54]. A wide range of commercially available antigen- and antibody-based assays is currently on the market, with varying levels of scientific evidence supporting their use in diagnosing IA. Most clinically validated assays carry CE IVD marking, allowing their implementation in routine diagnostic workflows and indicating compliance with established regulatory requirements. Research-use-only (RUO) assays are also available; however, their use in clinical decision-making is generally not recommended, as they lack regulatory approval, standardised clinical validation, and evidence-based performance characteristics required for routine diagnostic application [3,55]. A selection of the most commonly used antigen and antibody detection kits is summarised in Table 1.

### 4.1. Galactomannan

Galactomannan (GM), a polysaccharide constituent of the Aspergillus cell wall, is the most widely used antigenic biomarker in clinical practice. GM enzyme immunoassays (EIA) are incorporated into international consensus definitions and are recommended for diagnosis and monitoring in high-risk patients [1,56,57].

#### 4.1.1. Diagnostic Performance

Meta-analyses in haematology populations report serum GM sensitivity of approximately 70–80% and specificity of 80–90% at commonly used optical density index (ODI) cut-offs (≥0.5) [54,56]. Sensitivity increases when two consecutive positive samples are required, which enhances specificity but may delay diagnosis [54,56]. In contrast, GM detection in BALF shows higher sensitivity—often exceeding 80%—with comparable specificity, making it particularly valuable in suspected invasive pulmonary aspergillosis [1,23].

#### 4.1.2. Matrix Considerations

Test performance varies significantly by specimen type. Serum assays are convenient but less sensitive in non-neutropenic hosts and in patients receiving mould-active prophylaxis or therapy [1,13]. BALF, obtained during bronchoscopy, more accurately reflects local fungal burden and consistently yields superior sensitivity, albeit at the cost of an invasive procedure [23,54].

#### 4.1.3. Factors Influencing Test Performance

Several factors may affect GM assay interpretation [1,54,58,59,60]:

- Antifungal exposure—mould-active agents such as voriconazole or posaconazole may suppress antigen release, resulting in false-negative results.
- Cross-reactivity—other moulds (e.g., *Fusarium*, *Penicillium*) and certain β-lactam antibiotics may cause false-positive results, although improved assay specificity has reduced this issue.
- Renal replacement therapies—haemodialysis and plasma exchange may alter circulating antigen levels and complicate interpretation.
- Exogenous polysaccharide-containing products—false-positive GM results may occur after exposure to non-fungal sources of galactomannan or structurally related polysaccharides. Reported causes include glucose-containing intravenous fluids, administration of intravenous immunoglobulins, and plant-derived haemostatic materials used during surgery.

#### 4.1.4. Clinical Utility and Monitoring

Beyond diagnosis, GM kinetics may assist in treatment monitoring. Declining GM indices correlate with favourable outcomes in some studies, while persistently elevated or rising values may indicate treatment failure or disease progression. However, standardised thresholds for monitoring remain debated [11,54,61].

### 4.2. β-D-Glucan

(1→3) β-D-glucan (BDG) is a cell wall component of most fungal species. BDG assays detect circulating glucan in serum and have a high negative predictive value, making them useful for ruling out invasive fungal infection. However, BDG lacks specificity for Aspergillus and may be elevated in other fungal diseases, including candidiasis and *Pneumocystis* infection, limiting its standalone diagnostic value [3,13,54].

### 4.3. Lateral Flow and Point-of-Care Antigen Assays

Lateral flow assays (LFAs) targeting *Aspergillus*-specific antigens provide rapid, point-of-care testing with results available within 30–60 min. Meta-analyses report pooled sensitivity of approximately 65–80% and specificity of 85–95%, with superior performance in BALF compared with serum. LFAs are simple to perform and suitable for decentralised laboratories or settings without specialised immunoassay platforms [23,54,59,62].

### 4.4. Emerging Antigenic Biomarkers

Research into novel antigenic biomarkers for aspergillosis is ongoing, with the aim of improving diagnostic specificity, enabling species differentiation, and facilitating non-invasive detection [19,54,62]. Among the approaches under investigation are mannan and species-specific glycoproteins unique to Aspergillus, as well as multiplex antigen panels that combine several targets within a single assay to enhance sensitivity and discriminatory capacity. In addition, volatile organic compounds (VOCs) in exhaled breath represent a promising non-invasive diagnostic strategy, although current evidence remains preliminary.

More recently, attention has shifted toward host-derived and fungal metabolic biomarkers. Pentraxin 3 (PTX3), a soluble pattern-recognition molecule released by myeloid and endothelial cells during invasive fungal infection, together with fungal metabolites such as gliotoxin and its derivative bis(methylthio)gliotoxin (bmGT), and Aspergillus metallophores—small metal-chelating compounds involved in nutrient acquisition—have all emerged as potential diagnostic targets [63]. However, their performance and clinical applicability vary considerably.

Among these, PTX3 currently demonstrates the most consistent diagnostic potential. In bronchoalveolar lavage (BAL) fluid, PTX3 achieved a sensitivity of 86.3% and specificity of 82.5% [64], while in lung transplant recipients, BAL PTX3 concentrations >319 pg/mL were associated with a 4.5-fold increased likelihood of invasive aspergillosis [65]. Furthermore, combining PTX3 with other inflammatory markers, such as S100A12, may further improve diagnostic accuracy [66].

In contrast, evidence for fungal metabolites remains inconsistent. Vidal-García et al. (2016) [67] reported that bmGT outperformed galactomannan, demonstrating higher sensitivity and positive predictive value, with combined testing achieving a PPV of 100% and NPV of 97.5% in a cohort of 79 patients. However, Mercier et al. (2019) [68] concluded that gliotoxin and bmGT are not reliable diagnostic biomarkers. Experimental data further suggest that gliotoxin detectability may depend on host immune status, being detectable in 71% of sera from neutropenic mice but absent in steroid-treated models [69].

Aspergillus metallophores represent a novel and promising class of biomarkers; however, current evidence remains limited and largely exploratory [63], and their clinical utility will depend on validation in well-designed human studies.

Overall, PTX3 appears to hold the greatest near-term diagnostic potential, whereas bmGT, gliotoxin, and metallophores require further investigation before they can be integrated into routine clinical practice.

### 4.5. Non-Standard Specimen Types

Antigen testing in cerebrospinal fluid (CSF) remains largely investigational due to low pathogen burden and poor sensitivity in central nervous system aspergillosis. Similarly, urine antigen detection has been explored but is not validated for routine clinical use [19,54].

### 4.6. Antigen Testing in Allergic Aspergillosis

In allergic bronchopulmonary aspergillosis (ABPA), antigen assays have limited diagnostic value. Immunological markers—including total IgE and *Aspergillus*-specific IgE and IgG—remain the cornerstone of diagnosis, reflecting host sensitisation rather than fungal burden [2,6].

### 4.7. Practical Considerations

Antigen assays are rapid, widely available, and easily integrated into routine clinical workflows. However, results must always be interpreted in clinical context, taking into account host factors, specimen type, prior antifungal exposure, and the potential for cross-reactivity [16,54,62]. Antigen-based methods such as GM remain diagnostically valuable in IA due to their relatively good sensitivity and specificity, as well as their ability to provide timely diagnostic information. However, their role in early detection may be limited in certain settings. Rather than consistently establishing the initial diagnosis, GM more often supports a clinically suspected infection, highlighting its complementary role [70,71].

## 5. Molecular Diagnostics: Real-Time PCR, Resistance Detection, and Next-Generation Sequencing

Molecular diagnostics have substantially improved the detection of *Aspergillus* spp., particularly in patients in whom culture-based methods lack sensitivity. Advances in assay standardisation, resistance detection, and high-throughput sequencing have expanded the diagnostic armamentarium, although challenges remain regarding interpretation, accessibility, and clinical integration [20,42,63].

### 5.1. Real-Time PCR

Real-time PCR assays for Aspergillus DNA have significantly enhanced diagnostic sensitivity. Standardisation efforts by the European Aspergillus PCR Initiative have improved assay reproducibility and inter-laboratory comparability. Meta-analyses demonstrate that PCR, particularly when performed on BALF, provides higher sensitivity than culture and improves diagnostic performance when combined with GM testing [23,42,54].

In blood samples, PCR sensitivity is generally lower than in BALF but may allow earlier detection in selected high-risk populations. Plasma cell-free DNA PCR represents a promising non-invasive approach, with potential for detection before radiological progression. Despite these advantages, PCR has limitations [42,62,72]. Detection of fungal DNA does not distinguish colonisation from invasive disease, and quantitative thresholds vary across platforms. Antifungal therapy may reduce detectable DNA levels, and strict contamination control is essential to avoid false positives. Validated protocols and quality assurance measures are therefore critical for reliable implementation [42,62,72]. A wide range of commercially available real-time PCR kits is currently on the market, with varying levels of scientific evidence supporting their use in the diagnosis of aspergillosis. Most clinically validated kits for IA carry CE-IVD marking, making them suitable for routine diagnostic use. Research-use-only (RUO) kits are also available, but they are generally not appropriate for clinical decision-making [3,55]. A list of the most commonly used kits is provided in Table 2.

Interpretation of the comparative data indicates that AsperGenius^®^ provides the most balanced diagnostic performance, combining relatively high sensitivity with consistently high specificity, making it well suited for routine clinical use. In contrast, MycoGENIE^®^ demonstrates higher sensitivity in several studies and may therefore be advantageous as a screening tool, although this comes at the expense of reduced specificity and a higher likelihood of false-positive results [42,73,74]. Conversely, Fungiplex^®^ Aspergillus exhibits higher specificity but lower sensitivity, supporting its use primarily as a confirmatory (rule-in) assay in patients with a high pre-test probability of IA [42,73]. Other CE-IVD assays (e.g., gb MICRO, GeneProof^®^, AspID^®^) are available but currently lack robust, independent clinical performance data, which limits their comparability and broader clinical interpretation [42].

### 5.2. Molecular Detection of Resistance

Triazole resistance in *A. fumigatus* is an increasingly important global concern, typically affecting 1–10% of clinical isolates in many centres, although local prevalence may exceed 20% and reach ≥60% in certain Asian settings [75,76]; in Belgium and the Netherlands, rates of approximately 7–8% have been reported [77,78]. Most resistant isolates harbour *cyp51A* mutations, particularly the environmentally associated TR_34_/L98H and TR_46_/Y121F/T289A alterations, although their distribution varies geographically (Sharpe et al. 2018) [32,77,78,79,80]. However, a substantial proportion of resistant isolates—often exceeding 50% in some cohorts—lack detectable *cyp51A* mutations, indicating alternative resistance mechanisms, including mutations in *hmg1* and *hapE*, overexpression of *cyp51A/B*, upregulation of efflux transporters, and broader polygenic contributions [81,82,83,84,85].

Molecular diagnostics play an increasingly important role in the detection of resistance-associated mutations, particularly those in *cyp51A* that confer azole resistance. Detection strategies integrate phenotypic AFST with targeted genotyping, while PCR-based assays and pyrosequencing enable rapid identification of common resistance mutations, including TR_34_/L98H and TR_46_/Y121F/T289A, directly from clinical specimens (Table 2), often providing earlier guidance for antifungal therapy than conventional methods samples [13,30,32,79,86,87]. This is especially relevant in settings with a high prevalence of environmentally driven resistance [13,30,32]. Early identification of resistant strains supports timely therapeutic modification and informs antifungal stewardship [13,30,32]. Nevertheless, as many resistant isolates are *cyp51A*-wild-type or harbour atypical alterations, comprehensive approaches—such as whole-genome or targeted sequencing of *hmg1*, *hapE*, efflux pump genes, and other components of the ergosterol pathway, together with gene expression analyses—are increasingly required to fully capture the heterogeneous and evolving landscape of triazole resistance [78,79,88].

### 5.3. Next-Generation Sequencing

Next-generation sequencing (NGS), particularly metagenomic NGS, enables unbiased detection of fungal, bacterial, and viral DNA in a single assay. In selected cohorts, reported sensitivity and specificity for invasive fungal infections often exceed 85–90%, although performance varies depending on sample type and analytical approach [20,72,89]. NGS is particularly valuable in diagnostically challenging cases, such as atypical presentations, mixed infections, or persistently negative conventional tests [20,72]. However, several limitations restrict widespread clinical adoption [20,62,72]:

- High cost and limited availability.
- Bioinformatic complexity, requiring specialised expertise.
- Longer turnaround times compared with targeted molecular assays.
- Lack of standardised diagnostic thresholds, complicating interpretation.
- Potential detection of environmental or colonising organisms, which may not represent true invasive disease.

Despite these challenges, NGS continues to evolve, with improvements in sequencing speed, cost efficiency, and analytical pipelines expected to enhance clinical utility.

### 5.4. Emerging Molecular and Host-Based Approaches

Several novel molecular approaches are under investigation [20,42,62,72,90]:

- Digital PCR offers improved quantification and sensitivity compared with conventional real-time PCR, potentially enabling more precise fungal load monitoring.
- Targeted amplicon sequencing may allow simultaneous species identification and resistance profiling within a single workflow.
- Host response biomarkers, including transcriptomic and cytokine signatures, are being explored as adjunctive tools for distinguishing colonisation from invasive disease. Integration of host and pathogen data may represent a future direction for improving diagnostic specificity.

Molecular diagnostics have become indispensable in the evaluation of suspected aspergillosis. Real-time PCR enhances early detection, resistance assays provide actionable therapeutic information, and NGS offers broad, unbiased pathogen identification [32,42,72]. Continued progress will depend on harmonised protocols, improved accessibility, and integration of molecular data with clinical and radiological findings [21,62].

## 6. Combination Strategies

Because no single laboratory assay achieves optimal sensitivity and specificity across all patient populations and clinical contexts, combined diagnostic strategies are central to contemporary management of aspergillosis [13,22,90]. The rationale for multimodal diagnostics lies in the complementary strengths and limitations of individual tests: culture offers high specificity but low sensitivity; GM provides relatively early detection but variable performance depending on host factors; PCR enhances sensitivity but may detect colonisation; and BDG offers high negative predictive value but limited pathogen specificity [3,42,54,62]. Accordingly, integrating these modalities represents the most reliable strategy for both confirming and excluding disease, as consistently highlighted in the literature.

### 6.1. Combined Biomarker Approaches

Numerous studies have shown that simultaneous or sequential use of GM and PCR improves diagnostic accuracy compared with either method alone [16,42,54,62]. In high-risk haematological patients, combining serum GM and Aspergillus PCR increases sensitivity while maintaining acceptable specificity, particularly when two consecutive positive results are required [16,22,23,56]. This approach reduces false positive rates and improves positive predictive value in low-prevalence settings [13,54].

BALF-based combinations are especially effective in suspected invasive pulmonary aspergillosis [13,22,23]. Concurrent detection of GM and Aspergillus DNA in BALF significantly increases the likelihood of true invasive disease compared with a single positive test [42,54,62]. Conversely, dual negative BALF GM and PCR results provide strong negative predictive value and may support early discontinuation of empirical antifungal therapy [13,22,23].

BDG may further enhance sensitivity when added to GM and PCR in selected populations, although its lack of species specificity requires cautious interpretation. In centres with high background rates of candidiasis or other invasive mycoses, BDG positivity may not reliably distinguish Aspergillus infection [13,15,22,54,62].

### 6.2. Impact on Antifungal Stewardship

One of the most important clinical implications of combination strategies is their role in antifungal stewardship. Biomarker-driven approaches, such as serial GM and PCR testing, have shown potential to reduce unnecessary empirical antifungal therapy in neutropenic patients without increasing mortality [13,22,62,91]. These pre-emptive strategies aim to balance early targeted treatment with avoidance of unnecessary antifungal exposure, toxicity, drug interactions, and resistance selection [13,22,62,91].

Serial biomarker testing also improves the temporal resolution of disease evolution. Rising GM indices or increasing PCR fungal load may precede radiological progression and assist in early therapeutic escalation. Conversely, declining biomarker levels may support assessment of treatment response, although standardised thresholds for monitoring are still under evaluation [22,54,61,62].

### 6.3. Integration with Radiology and Clinical Risk Stratification

Laboratory diagnostics must be interpreted alongside radiological findings, particularly high-resolution computed tomography (CT) of the chest [3,7,13]. Classic radiological signs such as the halo sign or air-crescent sign increase the pre-test probability of invasive pulmonary aspergillosis, thereby improving the positive predictive value of laboratory assays. Conversely, in low-risk populations without compatible imaging findings, isolated positive biomarkers have reduced specificity [3,13,22,23,25].

Risk-adapted diagnostic algorithms are increasingly advocated. In neutropenic haematology patients, serial serum screening (GM ± PCR) is commonly employed [22,54,61]. In non-neutropenic intensive care unit (ICU) patients, BALF-based testing is generally preferred due to lower serum assay sensitivity. Such stratified approaches optimise diagnostic yield while limiting unnecessary investigations [2,7,13,21,28].

### 6.4. Combination Strategies in Allergic Aspergillosis

In allergic bronchopulmonary aspergillosis (ABPA), diagnosis relies on a multimodal framework that combines clinical features, total IgE, *Aspergillus*-specific IgE and IgG, peripheral eosinophilia, and characteristic radiological abnormalities [2,6,92,93,94]. Sensitivity and specificity odd Aspergillus EIA is in general 83.8 to 92.9% and 92.9 to 99.3%. respectively [93,94]. A sudden rise in quantitative result can imply therapy failure. No single serological parameter is sufficient for diagnosis. Serial measurement of total IgE is also useful for monitoring disease activity and treatment response [2,6,57,93,94]. Emerging research suggests that combining conventional immunological markers with molecular detection of airway colonisation may further refine diagnostic precision, although this remains investigational [20,21,72].

Combination diagnostic strategies represent the current standard of care for IA in high-risk populations. By integrating complementary laboratory modalities with clinical and radiological assessment, these approaches enhance sensitivity, improve specificity, and support antifungal stewardship [7,13,21,22]. Continued refinement of algorithm-based and data-driven diagnostic frameworks is likely to further improve precision and patient outcomes [7,21,91].

### 6.5. Personalised and Combined Diagnostic Approaches in Aspergillosis

As shown, combined diagnostic approaches are increasingly recognised as essential for optimal diagnosis of aspergillosis, with contemporary strategies progressively incorporating elements of individualisation based on patient-specific risk profiles and clinical context [63,95,96]. This paradigm, consistently supported by recent literature, reflects the limitations of single diagnostic assays and the need to integrate complementary modalities within clinically meaningful frameworks [1,7,16].

Alongside multimodal testing, there is a growing shift towards personalised diagnostic strategies. Currently, individualisation is primarily achieved through risk-adapted test selection and phenotype-specific diagnostic algorithms, rather than fully individualised biomarkers [4,63,95,96]. Distinct diagnostic pathways are applied across patient populations, including haematological and transplant recipients, critically ill non-neutropenic patients, and those with chronic or allergic forms of aspergillosis. In these settings, the choice, timing, and interpretation of diagnostic tools—such as galactomannan, β-D-glucan, PCR, serology, and imaging—are tailored to host factors, pre-test probability, antifungal exposure, and specimen type.

Early elements of precision diagnostics are already being incorporated into clinical practice [4,97]. Molecular assays provide species identification and detection of azole-resistance mutations, supporting targeted antifungal therapy, while metagenomic next-generation sequencing is increasingly used in selected high-risk or diagnostically complex cases to enable comprehensive pathogen detection, including mixed infections [98,99,100].

The concept of personalised “theranostics”, as introduced by Oliveira-Coelho et al. (2015) [98], further underscores the potential of integrating individual patient characteristics into predictive and clinically applicable diagnostic models. Future developments are expected to expand this approach through the incorporation of host genetic susceptibility markers and multi-omics data, enabling more precise risk stratification and prediction of disease progression and treatment response [64,99,101].

However, despite these advances, personalised diagnostics in aspergillosis remain in development. As highlighted by Jenks et al. (2019) [22], further validation of emerging biomarkers—particularly in non-haematological populations—is still required before widespread clinical implementation can be achieved.

Overall, current evidence supports a multimodal and increasingly individualised diagnostic paradigm, in which the strategic combination of complementary tests is adapted to the individual patient, while future progress will depend on the validation and integration of novel biomarkers into standardised clinical frameworks.

## 7. Conclusions

Combination diagnostic strategies now represent the standard of care in high-risk populations. Integrating culture, antigen detection, molecular assays, radiological findings, and clinical risk stratification enhances diagnostic precision, supports antifungal stewardship, and improves patient outcomes. Future progress will depend on harmonised molecular protocols, rapid and accessible resistance detection, cost-effective diagnostic algorithms, and the incorporation of host response biomarkers to better distinguish colonisation from true invasive disease. International clinical guidelines remain a critical foundation for the diagnosis and management of aspergillosis, providing evidence-based diagnostic algorithms and standardised care pathways. However, an individualised diagnostic and therapeutic approach is essential, as patients are clinically heterogeneous and often present with complex comorbidities, variable environmental exposures, and differing risk profiles. Local epidemiology and antifungal resistance patterns may also require adaptation of guideline recommendations. Diagnostic and treatment decisions should therefore integrate guideline-based strategies with host factors, available microbiological and radiological data, and the local clinical context to optimise the timing and selection of antifungal therapy and ultimately improve patient outcomes.

## Figures and Tables

**Table 1:** Overview of commercially available antigen and antibody detection kits used in the diagnosis of aspergillosis. The information was obtained from the respective manufacturers’ websites. BALF—bronchoalveolar lavage fluid; IVD—in vitro diagnostics; RUO—research use only; CLIA—chemiluminescent immunoassay; ELISA—enzyme-linked immunosorbent assay; FEIA—fluorescence enzyme immunoassay; LAL—*Limulus* amebocyte lysate assay; LFA—lateral flow assay.

Kit	Manufacturer	Method	Targets	Sample Type	Status
Aspergillus GM Rapid Test	Abbexa, Cambridge, UK	Lateral flow	Galactomannan	Serum, plasma, whole blood	RUO
Aspergillus GM Lateral Flow Assay (LFA)	Immy, Norman, OK, USA	Lateral flow	Galactomannan	BALF, serum	IVD
Aspergillus Lateral Flow Device (LFD)	OLM Diagnostics, Newcastle upon Tyne, UK	Lateral flow	*Aspergillus* antigen (JF5 epitope)	BALF, serum	IVD
Fungitell^®^ Assay	Associates of Cape Cod, East Falmouth, MA, USA	Colorimetric (LAL-based)	(1→3)-β-D-glucan	Serum	IVD
FungiXpert^®^ (1→3)-β-D-glucan	Genobio, Tianjin, China	CLIA	(1→3)-β-D-glucan	Serum, BALF	IVD
FungiXpert^®^ Aspergillus GM ELISA	Genobio, Tianjin, China	ELISA	Galactomannan	Serum, BALF	IVD
FungiXpert^®^ Aspergillus IgG CLIA	Genobio, Tianjin, China	CLIA	*Aspergillus*-specific IgG	Serum	IVD
ImmunoCAP Aspergillus IgG/IgE	Thermo Fisher Scientific, Waltham, MA, USA	FEIA	*Aspergillus*-specific IgG/IgE	Serum	IVD
Platelia™ Aspergillus EIA	Bio-Rad, Hercules, CA, USA	ELISA	Galactomannan	Serum, BALF	IVD
QuicGM™ Aspergillus LFA	Dynamiker, Tianjin, China	Fluorescent LFA	Galactomannan	Serum, BALF	IVD
VirClia^®^ Aspergillus GM Monotest	Vircell, Granada, Spain	CLIA	Galactomannan	Serum	IVD

**Table 2:** Overview of commercially available real-time PCR kits for the diagnosis of aspergillosis. The information was obtained from the respective manufacturers’ websites. BALF—bronchoalveolar lavage fluid; CSF—cerebrospinal fluid; IVD—in vitro diagnostics; RUO—research use only.

Kit	Manufacturer	Targets	Sample Type	Status
AsperGenius^®^	PathoNostics, Maastricht, The Netherlands	*Aspergillus* spp., *A. fumigatus*, *A. flavus* *	BALF, serum, plasma, CSF (not validated)	IVD
AspID^®^ Aspergillus qPCR	LionDx, Venice, Italy	*Aspergillus* spp., *A. terreus*	BALF, serum, plasma	IVD
Fungiplex^®^ Aspergillus	Bruker, Billerica, MA, USA	*A. fumigatus*, *flavus*, *niger*, *terreus* *	BALF, serum, plasma	IVD
gb MICRO Aspergillus	Generi Biotech, Hradec Králové, Czech Republic	*A. fumigatus*, *flavus*, *niger*, *terreus*	Not specified	IVD
GeneProof^®^ Aspergillus PCR Kit	GeneProof, Brno, Czech Republic	*Aspergillus* spp.	Serum, BALF	IVD
iQ-Check Aspergillus	Bio-Rad, Hercules, CA, USA	*A. fumigatus*, *flavus*, *niger*, *terreus*	Non-clinical samples (e.g., cannabis products)	RUO
MycoGENIE^®^	Ademtech, Pessac, France	*Aspergillus* spp. ± *Mucorales* *	Serum, lower respiratory tract samples, tissue biopsies	IVD

* The kit or its corresponding variant also enables the detection of the most common mutations associated with clinical azole resistance.

## Data Availability

No new data were created or analyzed in this study. Data sharing is not applicable to this article.
